# Biomechanical Factors Associated with Intraindividual Differences in Running Economy Across Advanced Footwear Technology Models in Long-Distance Runners

**DOI:** 10.1186/s40798-026-01005-0

**Published:** 2026-03-20

**Authors:** Dominik Fohrmann, Marcelle Schaffarczyk, Carolin Menge, Steffen Willwacher, Alberto Sanchez-Alvarado, Thomas Gronwald, Karsten Hollander

**Affiliations:** 1 Institute of Interdisciplinary Exercise Science and Sports Medicine, MSH Medical School Hamburg, Am Kaiserkai 1, 20457 Hamburg, Germany; 2 Institute of Sport Science, Julius-Maximilians-Universität Würzburg, Würzburg, Germany; 3 Institute for Advanced Biomechanics and Motion Studies, Offenburg University of Applied Sciences, 77652 Offenburg, Germany; 4 Sports Medicine and Sports Orthopaedics, University Outpatient Clinic, University of Potsdam, 14476 Potsdam, Germany

**Keywords:** Carbon fiber plate, Oxygen cost, PEBA, Super shoes

## Abstract

**Background:**

Advanced footwear technology (AFT) can enhance long-distance running performance by improving running economy (RE). However, the range of the individual improvements is large. Different intra-individual responses in running biomechanics may account for some of the variation. Thus, this randomized within-subject crossover study aimed to identify biomechanical factors associated with changes in RE when running with different AFT models.

**Methods:**

Twenty-two trained long-distance runners (50% female) ran multiple 5-minute running bouts at their season’s best marathon pace (15.0 ± 2.3 km⸱h^− 1^) while wearing three standardized AFT models (Nike Air Zoom Alphafly Next% 2, Puma Fast-R Nitro Elite v1, Asics Metaspeed Sky+). During each condition, gas exchange data and three-dimensional kinematics and spatiotemporal variables were acquired. RE was determined as the energetic cost of transport. We used two complementary model selection strategies (Akaike Information Criterion model averaging and least absolute shrinkage and selection operator) to identify biomechanical parameters associated with intra-individual differences in RE, and a repeated measures ANOVA to compare RE between shoes at the group level.

**Results:**

Across shoe conditions, shorter ground contact time was significantly associated with lower energetic cost of transport (β = 0.025, 95% CI [0.010, 0.040], t(42) = 3.33, *p* = 0.002), reflecting a ~ 1% improvement in RE per 4 ms decrease. We did not find group-level differences in RE between shoe conditions (*p* = 0.246).

**Conclusions:**

AFT models that reduced runners’ individual ground contact time were associated with improved RE. This effect appears to depend on the individual athlete-shoe interaction since no single AFT model stood out as an overall optimum. These findings can help determine optimal footwear for athletes. Future studies should investigate the interaction of AFT properties and individual biomechanics to identify further RE improvements through footwear individualization.

**Supplementary Information:**

The online version contains supplementary material available at 10.1186/s40798-026-01005-0.

## Key Points

- This study revealed that shorter ground contact time was a robust predictor of lower energetic cost of transport (≈ 1% per 4 ms decrease) in experienced long-distance runners across three standardized AFT models.
- No significant differences in running economy were detected among AFT shoe conditions at the group level.
- Our findings may aid individualized footwear selection.

**Supplementary Information:**

The online version contains supplementary material available at 10.1186/s40798-026-01005-0.

## Background

Shoes are probably the most important piece of equipment for competitive runners as they are at the impact-generating interface between the athlete and the ground. Recent technological advances in materials and design, among other potential factors, have led to repeatedly surpassed world records in the long-distance running disciplines, most prominently the marathon distance [1, 2]. These advanced footwear technology (AFT) shoes are comprised of highly compliant and thick midsole materials and typically a rigid, curved stiffening element integrated into the midsole. They have not only become the standard choice among elite runners but have also become popular among lower-caliber athletes [3, 4].

Previous research revealed an improved running economy (RE) of approximately 3–4% when running in AFT compared to less technologically advanced marathon running shoes [5–7]. RE is the body mass-specific rate of energy expenditure or oxygen uptake per distance covered at a constant submaximal speed [8–10]. It is strongly correlated to athletic performance in distance running [11]. Based on the empirical findings by Hoogkamer et al. [6] and the modeling approach by Kipp et al. [12], a 4% improvement in RE — as observed with advanced footwear technology — is predicted to result in approximately a 3.4% increase in running velocity at sub-elite marathon speeds (~ 16 km⸱h^− 1^), considering the non-linear relationship between oxygen uptake and velocity as well as the additional cost of air resistance. An illustrative example highlighting the relevance of RE for long-distance running performance is the progress of the former female marathon world record holder Paula Radcliffe who reduced her oxygen uptake while running at 16 km·h^− 1^ by 2.8% and improved marathon performance by 2.4% without changes in VO_2_max [13].

Despite the average improvements in RE, individual changes ranged from 1.6 to 6.3% [6] or even − 1.1 to 9.7% [5, 14]. Measurement errors undoubtedly account for some of this variation [15], but inter-individual variance in response to different types of AFT affecting RE may account for a large part of it. By definition, AFT models share the same general design features [16]. However, manufacturer- and model-specific design choices, e.g., in terms of midsole material density and longitudinal bending stiffness, may lead to different responses for individual runners [17]. In fact, a recent framework outlines the distinct pathways by which footwear impacts RE [18]. There is a direct pathway reflecting the – athlete-independent – influence of the material properties. The technologically advanced midsole foams showed superior resilience, i.e., energy return capacity, when compared with traditional running shoes [6]. Energetic calculations, however, revealed that this effect alone does not entirely explain the overall metabolic savings [19]. Thus, interactive effects between footwear features, such as longitudinal bending stiffness or compression characteristics, and the athlete’s individual biomechanics must be considered. In the above-mentioned framework, this is reflected by a secondary, indirect pathway. This pathway is thought to account for a certain amount of inter-individual and between-shoe variation in previous studies.

To better understand these inter-individual differences, it is essential to examine how AFT modifies running biomechanics. Group-level studies indicate that AFT use (versus use of traditional shoes) alters ankle and metatarsophalangeal (MTP) joint kinetics and kinematics, but not knee and hip mechanics [19]. Specifically, running in AFT was correlated with decreased negative work in the ankle and MTP joint and decreased positive work in the ankle joint, in combination with decreased peak dorsiflexion angles and moments in both MTP and ankle joints when compared to running in traditional running shoes [19]. Another study revealed that improved RE in an AFT was related to increased stride length accompanied by increased vertical oscillation, and decreased ankle plantarflexion velocity [7]. However, these biomechanical correlates are mostly derived from averaged group data, leaving the role of individual biomechanics in mediating RE responses largely unresolved.

A first step towards addressing within-subject differences was recently made by van Hooren et al. [20], who investigated whether individual running biomechanics and anthropometrics mediate the relationship between AFT use and RE. Their study provided initial evidence of the role of biomechanics and anthropometrics in explaining inter-individual variability. However, the magnitude of this mediating effect and the specific biomechanical determinants remain unclear. Moreover, their analysis compared two AFT shoes against a neutral control shoe and did not directly compare various AFT models. This leaves the question open whether different AFT designs interact differently with running biomechanics to modulate RE. No study to date has investigated individual running biomechanics across various AFT models and linked them to changes in RE.

Understanding how individual running biomechanics interact with and respond to different types of AFT is crucial for elucidating the mechanisms by which AFT influences RE. These interactions may enhance or impair RE depending on the biomechanical compatibility between the runner and the shoe. A within-subject approach is necessary to study athlete-shoe interactions and explain the range in RE changes seen in previous research. Therefore, the aim of this exploratory study was to identify biomechanical factors associated with intra-individual changes in RE when running in various AFT models using a within-subject approach. This will aid informed individualized shoe selection and advance understanding of performance determinants.

## Methods

### Participants

We recruited competitive long-distance runners (tier 2 to tier 4 according to [21]) from the German Athletics Federation, Hamburg Athletics Federation, and local clubs through personal contacts. The inclusion criteria encompassed an official season’s best time in the marathon, half-marathon, or 10 km equivalent to 300 points on the World Athletics “Scoring Table of Athletics” [22], age between 18 and 65 years, and no lower limb injuries in the three months prior to study participation. Ethics approval for the present study was granted by the local ethics committee of the MSH Medical School Hamburg (reference no.: MSH-2023/233). All participants gave written informed consent, and all testing and measurements were conducted in accordance with the principles of the most recent revision of the Declaration of Helsinki. Reporting of our results was informed by the Strengthening the Reporting of Observational Studies in Epidemiology (STROBE) statement [23].

### Protocol

**Fig. 1:**
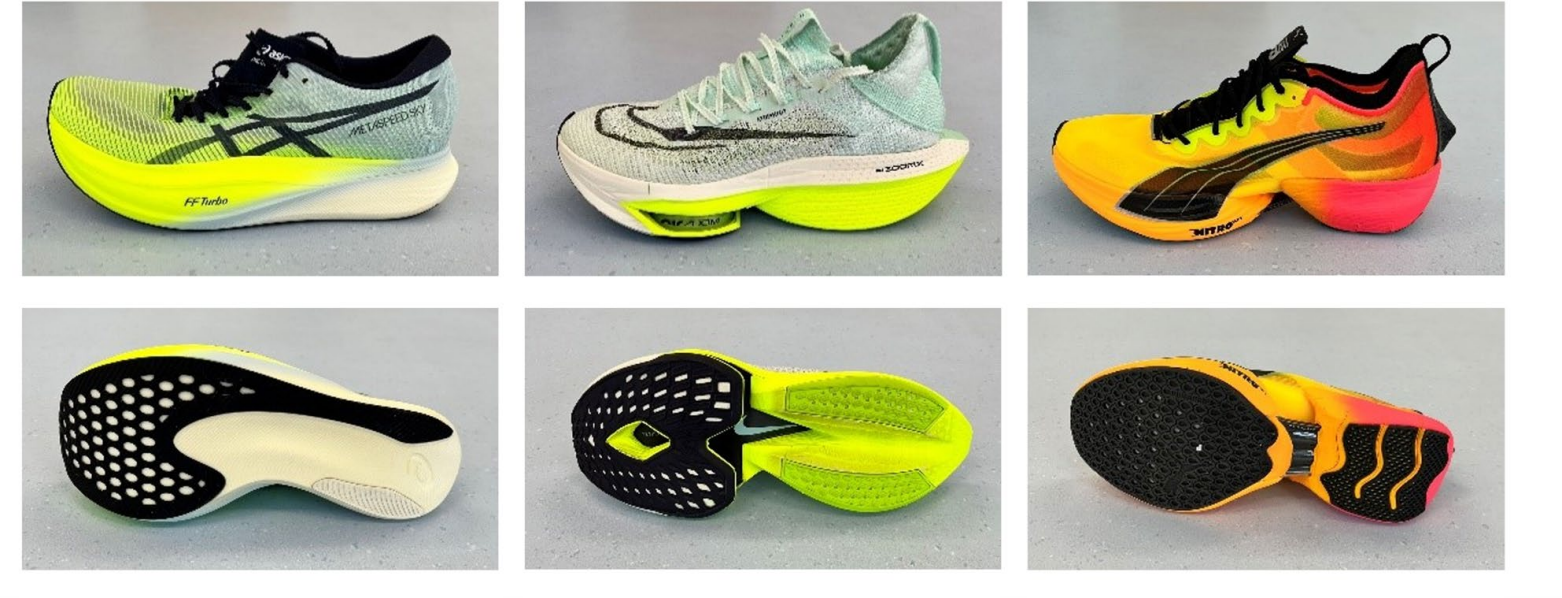
Standardized AFT models used in this study: Asics Metaspeed Sky+, Nike Air Zoom Alphafly Next% 2, Puma Fast-R Nitro Elite v1 (left to right)

Measurements took place in the university’s biomechanics laboratory during a single visit. Participants were provided with three different standardized AFT shoes: Nike Air Zoom Alphafly Next% 2 (NA), Puma Fast-R Nitro Elite v1 (PN), and Asics Metaspeed Sky+ (AM) (Fig. 1). While the PN and NA were available in “men” and “women” sizes, the AM was provided in a unisex size. The selection was based on the availability of three established running shoe brands. All models had features previously described in a formal definition of AFT [16]. Additionally, participants were allowed to bring one to three pairs of their own running shoes for their own reference, but these shoes were not included in the formal analysis here [24, 25]. After an individual ten-minute warm-up, participants completed four to six 5-minute running bouts in the different shoe conditions (depending on the number of running shoes brought along) at submaximal velocity in a randomized order. Each running bout was followed by a 5-minute resting period during which participants stood still upright, and shoes were changed with the help of the researchers. Running speed was determined using the athlete’s season’s best marathon time or by converting the season’s best performance over the half-marathon or 10 km distance into an estimated marathon time by multiplying those times by 2.11 or 4.66, respectively [26].

### Data Collection

All running bouts were conducted on a motorized treadmill instrumented with a pressure platform (FDM-T, h/p/cosmos, Nussdorf-Traunstein, Germany) at an inclination of 0.5% [27]. Breath-by-breath pulmonary gas exchange data were continuously recorded using a metabolic cart (Quark CPET, module A-670-100-005, COSMED Deutschland GmbH, Fridolfing, Germany; Omnia version 2.2) coupled with the Polar H10 sensor chest strap device (Polar Electro Oy, Kempele, Finland) to record the heart rate (HR; in beats per minute, bpm). Immediately after each running bout, the rating of perceived exertion was assessed using the Borg scale (scale of 6–20 [28]), and the perception of the comfort of the shoes was requested using a scale from 0 (‘not comfortable at all’) to 10 (‘maximally comfortable’) [29]. In addition, blood lactate concentration ([La]) was taken from capillary blood of the earlobe (20 µl) and analyzed after the testing session (Biosen C-Line Clinic analyser, EKF-diagnostic GmbH, Barleben, Germany).

A total of 42 spherical retroreflective markers were attached to the skin at the lower body and shoes according to an established set of anatomical landmarks [30]. The PN condition did not allow placing the posterior heel marker due to a “fin” as a design element at the heel cap (Fig. 1). As a solution, two markers were placed left and right of the fin, such that during post processing a virtual posterior heel marker could be calculated as the midpoint between those two. Kinematic data were captured during the last minute of each running bout using 12 cameras (300 Hz, Miqus Hybrid, Qualisys AB, Gothenburg, Sweden). Kinetic data were recorded in sync with the camera system with the pressure platform integrated in the treadmill (300 Hz, FDM-3i, Zebris Medical GmbH, Weitnau, Germany). We further measured the weight of each pair of shoes using a fine scale (TP-2000, SSR-Produkt GmbH & Co. KG, Oldenburg, Germany).

### Data Processing

From the continuous gas exchange measurements, we extracted the average oxygen uptake (VO_2_), respiratory exchange ratio (RER), respiratory frequency (RF) and HR over the last three minutes of each running bout. To confirm a steady rate of oxygen consumption, we fitted a linear regression over these 3-minute periods and verified that the slope was below the recommended threshold of 150 mL·min⁻¹, indicating minimal drift in VO₂ and thus steady-state conditions, as previously reported [20, 31]. Using the formula of Péronnet and Massicotte [32], we calculated the RE defined as energetic cost of transport (eCoT, in J⸱kg^− 1^⸱m^− 1^) as the primary outcome. Additionally, we calculated oxygen cost of transport (O_2_CoT, in ml⸱kg^− 1^⸱km^− 1^), normalized rate of oxygen uptake, and metabolic power, as these are frequently reported as RE parameters.

Kinematic data were post-processed using Visual3D (version 2023.12.1, C-Motions Inc., Germantown, MD, USA). Gaps in the trajectories were filled using a third order polynomial interpolation up to ten frames (i.e., 33ms). Raw trajectories were filtered using a 4th order recursive Butterworth filter with a cutoff frequency of 14 Hz [19]. Pelvis and lower limb joint kinematics of both sides were computed using Visual3D’s anthropometric model with six degrees of freedom joints. These signals were subsequently post-processed using custom Python scripts (version 3.10, python.org). If markers lost contact during the trials (25 of 109 measurements), only one side was analyzed. If both sides were available, we calculated the average of both sides. Gait events were calculated from the integrated pressure data (i.e. normal force) with a threshold of 20 N. The signals were then time-normalized and discrete values were computed for sagittal plane kinematics. Specifically, vertical pelvis oscillation was quantified over the full stride, whereas all joint-angle metrics (peak hip, knee, and ankle dorsiflexion; ankle flexion ROM; peak MTP dorsiflexion; and MTP flexion ROM) were analyzed during the stance phase of the support leg only. Additionally, spatiotemporal parameters were computed from the pressure data, namely, step frequency, ground contact time (GCT) and flight time. These parameters were shown to be closely related to RE or susceptible to changes in footwear in previous research [19, 33].

Material tests were conducted for the standardized AFT models using an electrodynamic material testing machine (LTM10, ZwickRoell GmbH & Co. KG, Ulm, Germany). A vertical force of 2000 N was applied under controlled conditions, analogous to a previously reported protocol [6]. The compression behavior of the sole construction was determined in both the rearfoot (stamp area: 97.71 cm^2^) and forefoot (stamp area: 39.65 cm^2^) areas (shoe size: US “men/unisex” 9).

**Table 1:** Sex-specific demographic data

	Male M ± SD (range)	Female M ± SD (range)	Total M ± SD (range)
Age (years)	32.2 ± 4.1 (26.4–39.2)	34.0 ± 8.0 (21.6–49.5)	33.1 ± 6.2 (21.6–49.5)
Height (cm)	179.0 ± 4.9 (170–186)	167.8 ± 5.9 (161–177)	173.4 ± 7.8 (161–186)
Weight (kg)	69.4 ± 6.5 (59.0–81.4)	57.3 ± 4.6 (48.6–64.2)	63.4 ± 8.3 (48.6–81.4)
BMI (kg⋅m^− 2^)	21.6 ± 1.7 (18.6–23.5)	20.4 ± 1.6 (17.7–22.3)	21.0 ± 1.7 (17.7–23.5)
Speed (km⋅h^− 1^)	16.5 ± 1.7 (14.5–19.5)	13.5 ± 1.8 (10.9–16.5)	15.0 ± 2.3 (10.9–19.5)
WA scoring points	745.7 ± 261.4 (417–1166)	777.3 ± 214.6 (385–1118)	761.5 ± 234.0 (385–1166)
Number of participants	11	11	22

* BMI * body mass index,* WA*  World Athletics

### Statistical Analysis

Statistical analyses were performed using R (version 4.3.0) in RStudio (version 2023.06, Posit Software, Boston, MA, USA). We calculated and reported means (M) and standard deviations (SD) for eCoT (primary outcome), O_2_CoT, oxygen uptake, metabolic power, the biomechanical parameters, and the remaining physiological parameters. Between-shoe differences in physio-logical and biomechanical variables, as well as shoe comfort, were analyzed using one-way repeated measures analyses of variance (ANOVA). Sphericity was assessed using Mauchly’s test for sphericity. When the sphericity assumption was violated, Greenhouse-Geisser corrections were applied. In cases of statistically significant results, pairwise post hoc T-tests were performed using Bonferroni correction. The level of significance was set to alpha = 0.05 for all statistical tests.

For the primary research question, we initially cluster-mean-centered (by participant) all biomechanical predictor variables and shoe mass and scaled them to their grand standard deviations to isolate within-athlete associations and improve numerical stability. Multicollinearity among predictors (all biomechanical variables) was assessed using variance inflation factors (VIFs) in a linear mixed-effects model. Because flight time, GCT, and step rate were highly correlated (VIF > 6), flight time was excluded a priori. GCT (VIF = 2.3) and step rate (VIF = 2.9) were retained.

To identify biomechanical variables associated with eCoT, we used two complementary model selection strategies. First, we applied a multimodel inference approach based on the corrected Akaike Information Criterion (AICc) [34]. Specifically, the biomechanical parameters and shoe mass as covariates were used as fixed effects in a linear mixed effects model to predict eCoT with a random intercept for the participant level with maximum likelihood estimation. All subsets of fixed effects were fit and ranked by AICc. Full model averaging (zero-method) was performed to obtain unconditional coefficient estimates and CIs for all predictors [35]. Variable importance was calculated as the sum of Akaike weights across the ΔAICc ≤ 4 set. An intercept-only mixed model (null model) was included in the candidate set. If it ranks among the competitive models (ΔAICc ≤ 4) with non-trivial weight, evidence for added predictors can be interpreted as weak [36]. Second, we applied least absolute shrinkage and selection operator (LASSO) mixed model regularization on the fixed effects using the glmmLasso package in R (version 1.6.3). This technique estimates coefficients under an L1 penalty which shrinks some coefficients to exactly zero, yielding a sparse set of predictors [37]. The penalty λ was chosen by AIC. Predictors identified by both AICc-based model averaging, and LASSO were considered robust. The model-averaged results were prioritized for interpretation, while the LASSO results served as a conservative cross-validation of predictor relevance. Unless otherwise indicated, we report the semi-standardized coefficients (β), representing change in eCoT (J⸱kg^− 1^⸱m^− 1^) per 1 SD change in the coefficient.

As a robustness check, we performed a leave-one-participant-out sensitivity analysis to evaluate the stability of the model coefficient estimates. Linearity and homoscedasticity assumptions were checked using residual plots. Normality of residuals was validated using Q-Q plots.

### Missing Data

For one participant, HR data had to be excluded due to poor signal quality (percent artifact > 5%), probably because of poor skin contact of the chest strap. Due to poor signal quality of the heel markers of the PN condition, ankle and MTP joint parameters could not be calculated for one participant. The participant was still included in the predictor analysis without using data imputation techniques, as the maximum likelihood estimation used in the linear mixed effects model handles data that are missing at random [38]. For the ANOVAs on ankle and MTP joint parameters, this participant was excluded.

## Results

### Participants' Characteristics and Descriptive Results

A total of 22 runners (50% female, age: 33.1 ± 6.2 years, BMI: 21.0 ± 1.7 kg·m^− 2^) completed the study. Sex-specific demographic data are listed in Table 1. The athletes’ season’s best performances were determined over the distances 10 km to marathon. These corresponded to average rating points of 762 ± 234 (female runners: 777 ± 215; male runners: 746 ± 261) according to the current World Athletics scoring table. These in turn correspond to marathon times of 3:09:32 h:min:s for female runners and 2:37:45 h:min:s for male runners. The mean values of [La] and RER were 2.08 ± 0.72 mmol⸱l^− 1^ and 0.86 ± 0.05, respectively. These values, together with the inspection of the magnitude of slopes over the 3-minute oxygen consumption, were indicative of a steady state of oxygen consumption. Values for eCoT reached 4.13 ± 0.30 J⸱kg^− 1^⸱m^− 1^, 4.13 ± 0.32 J⸱kg^− 1^⸱m^− 1^, and 4.16 ± 0.35 J⸱kg^− 1^⸱m^− 1^ for AM, NA, and PN, respectively. The percentage mean differences in eCoT between the individual best and worst performing AFT model were 2.71 ± 1.40% (range 0.57 to 6.03%). A linear mixed effects model detected no protocol-order effect on eCoT (F(2, 42) = 0.47, *p* = 0.629).

### Intraindividual Associations of Biomechanics and Running Economy

The main analysis revealed that, within participants, longer ground contact time (GCT) was associated with higher eCoT (model-averaged estimate = 0.031, 95% CI: 0.015, 0.048, z = 3.67, *p* < 0.001). Step rate and MTP peak dorsiflexion angle also showed high importance (sum of Akaike weights were 0.70 and 0.54), but their averaged effects were not statistically significant (both *p* > 0.231). These variables may contribute to RE variability but did not show consistent effects across participants. The remaining biomechanical variables showed low importance (sum of Akaike weights ≤ 0.38) and their model-averaged estimates were not significant (all *p* > 0.503; vertical pelvis oscillation: 0.005, 95% CI: -0.013, 0.023; peak hip flexion: 0.000, 95% CI: -0.004, 0.005; peak knee flexion: 0.000, 95% CI: -0.005, 0.006; peak ankle flexion: 0.001, 95% CI: -0.008, 0.011; ankle flexion ROM: 0.000, 95% CI: -0.006, 0.006; and MTP flexion ROM: -0.005, 95% CI: -0.022, 0.012).

**Fig. 2:**
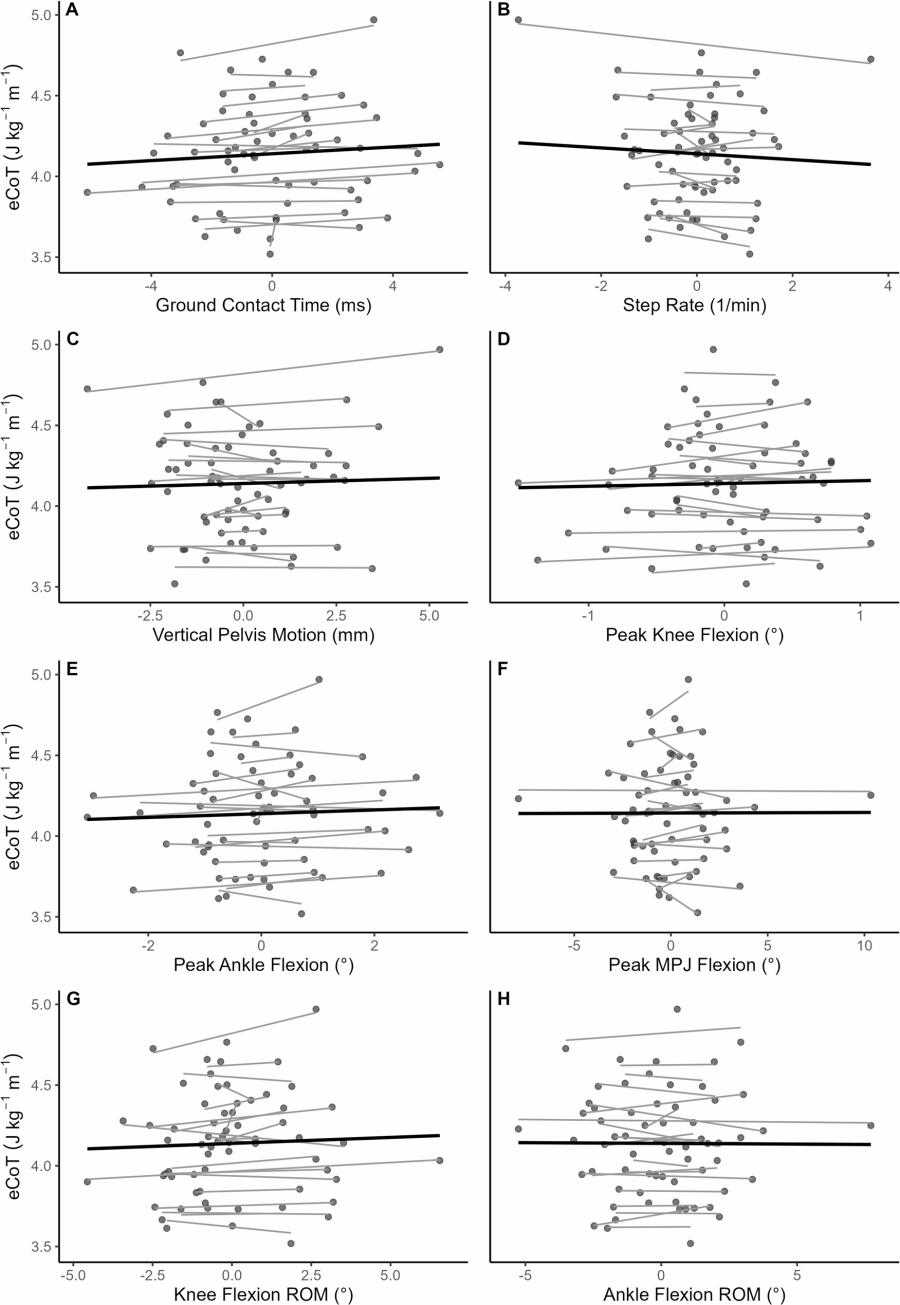
Isolated relationships of the biomechanical predictor variables and energetic cost of transport (eCoT). Only ground contact time (GCT, A) revealed a statistically significant relationship with eCoT, while step rate (B) and peak metatarsophalangeal joint (MTP) flexion (F) had a high variance importance in the full model averaging results. Predictor values are centered on the participant average. Thin lines represent single participant tendencies while the thick line represents the group-level trend.* ROM* range of motion

In this analysis, the shoe mass as a covariate was also not statistically significant (*p* > 0.397). The intercept-only model (null model) was not competitive (ΔAICc = 13.2), indicating that the observed effects were meaningful. The penalized LASSO analysis likewise shrank all coefficients (including that for shoe mass) to zero except GCT (β = 0.030). We therefore fitted a final linear mixed effects model with GCT as the only fixed effect and a participant random intercept, which showed a significant effect of GCT on eCoT (β = 0.025, 95% CI [0.010, 0.040], t(df = 42.0) = 3.33, *p* = 0.002). The leave-one-participant-out refits of this model yielded slopes in the range of 0.021 to 0.029, indicating robustness (Supplementary Figure S1). The relationships between all biomechanical variables and eCoT are depicted in Fig. 2.

### Between-Shoe Analysis

Repeated measures ANOVAs revealed no significant differences in eCoT between the three standard AFT shoe conditions (F(2,42) = 1.450, *p* = 0.246, η^2^_partial_ = 0.065) or in O_2_CoT (F(2,42) = 2.429, *p* = 0.100, η^2^_partial_ = 0.104), metabolic power (F(2,42) = 1.142, *p* = 0.329, η^2^_partial_ = 0.052) and normalized rate of oxygen uptake (F(2,42) = 2.23, *p* = 0.120, η^2^_partial_ = 0.096).

Of the biomechanical parameters, GCT (F(2,42) = 24.128, *p* < 0.001, η^2^_partial_ = 0.535), pelvis oscillation (F(2,42) = 4.243, *p* = 0.021, η^2^_partial_ = 0.168), peak ankle dorsiflexion angle (F(2,40) = 16.462, *p* < 0.001, η^2^_partial_ = 0.451), ankle flexion ROM (F(2,40) = 34.988, *p* < 0.001, η^2^_partial_ = 0.636), peak MTP dorsiflexion angle (F(2,40) = 12.44, *p* < 0.001, η^2^_partial_ = 0.383) and MTP flexion ROM (F(2,40) = 8.135, *p* = 0.003, η^2^_partial_ = 0.289) revealed overall differences between the three AFT models. Pairwise post hoc comparisons are reported in Table 2.

**Table 2:** Comparison of running economy, biomechanical, and physiological parameters between the standard shoe conditions

	AM	NA	PN	*p* value
	Mean ± SD	Mean ± SD	Mean ± SD	Main effect
Energetic cost of transport (J⋅kg− 1⋅m^− 1^)	4.13 ± 0.30	4.13 ± 0.32	4.16 ± 0.35	0.246
Oxygen cost of transport (ml⋅kg^− 1^⋅km^− 1^)	196.3 ± 14.7	196.4 ± 15.3	198.1 ± 16.4	0.100
Metabolic power (W⋅kg^− 1^)	17.2 ± 2.3	17.2 ± 2.3	17.3 ± 2.3	0.329
Oxygen consumption (ml⋅kg^− 1^⋅min^− 1^)	48.9 ± 6.3	48.9 ± 6.4	49.3 ± 6.5	0.120
Step frequency (min^− 1^)	172.3 ± 7.3	172.1 ± 7.4	172.1 ± 7.8	0.792
Ground contact time (s)	0.209 ± 0.022 ^NA, PN^	0.211 ± 0.022 ^PN^	0.214 ± 0.022	**< 0.001**
Flight time (s)	0.488 ± 0.023	0.487 ± 0.023	0.485 ± 0.024	0.170
Pelvis vertical oscillation (cm)	9.31 ± 1.09 ^NA^	9.48 ± 1.04	9.39 ± 1.15	**0.021**
Peak knee flexion (°)	41.9 ± 4.4	41.8 ± 4.6	42.0 ± 4.5	0.769
Peak ankle dorsiflexion (°)	14.4 ± 3.0 ^PN^	14.6 ± 2.9 ^PN^	16.4 ± 3.2*	**< 0.001**
Ankle flexion ROM (°)	37.2 ± 3.0 ^PN^	37.0 ± 3.0 ^PN^	40.5 ± 3.6*	**< 0.001**
Peak MTP dorsiflexion (°)	9.7 ± 1.9 ^NA, PN^	11.9 ± 3.8	13.3 ± 2.3*	**< 0.001**
MTP flexion ROM (°)	14.3 ± 2.4 ^PN^	12.7 ± 3.7 ^PN^	15.4 ± 2.5*	**0.003**
Heart rate (bpm)*	163.7 ± 10.9 ^PN^	163.1 ± 10.5 ^PN^	165.8 ± 11.5	**0.005**
Lactate (mmol⋅l^− 1^)	2.03 ± 0.66	2.06 ± 0.72	2.16 ± 0.8	0.100
RPE (Borg) (6–20)	12.7 ± 1.5	12.3 ± 1.3	12.9 ± 1.3	0.063
Respiratory exchange ratio ()	0.86 ± 0.05	0.86 ± 0.05	0.85 ± 0.04	0.291
Respiratory frequency (min^− 1^)	42.7 ± 7.6	42.5 ± 8.4	44.1 ± 7.5	**0.039**
Comfort (0–10)	7.2 ± 1.3	7.3 ± 1.6	6.9 ± 1.5	0.684

Statistically significant main effects (shoe condition) of the repeated measures ANOVA are printed in bold

*Data for 21 participants available only; ^NA^ significantly different from Nike, ^PN^ significantly different from Puma (both at *p* < 0.05)

*ROM* range of motion,* MTP * metatarsophalangeal joint,* RPE* rating of perceived exertion, AM Asics Metaspeed Sky+, NA Nike Air Zoom Alphafly Next% 2, PN Puma Fast-R Nitro Elite v1

With regard to the physiological metrics only HR (F(2,40) = 6.056, *p* = 0.005, η^2^_partial_ = 0.232) and RF (F(2,42) = 3.508, *p* = 0.039, η^2^_partial_ = 0.143) showed significant differences between the standard shoe conditions (HR: AM-NA: +0.6 bpm, *p* = 1.000; AM-PN: -2.1 bpm, *p* = 0.004; NA-PN: -2.7 bpm, *p* = 0.021; RF: AM-NA: +0.2 min-1, *p* = 1.000; AM-PN: -1.4 min-1, *p* = 0.068; NA-PN: -1.6 min-1, *p* = 0.124), while values for [La] (F(2,42) = 2.436, *p* = 0.100, η^2^_partial_ = 0.104), RER (F(2,42) = 1.257, *p* = 0.291, η^2^_partial_ = 0.056), RPE (F(2,42) = 2.956, *p* = 0.063, η^2^_partial_ = 0.123) and comfort (F(2,42) = 0.383, *p* = 0.684, η^2^_partial_ = 0.018) showed no statistically significant differences.

### Material Tests

The results of the material testing are presented in Table 3. NA exhibited the lowest stiffness (forefoot: 98.0 N·mm^− 1^, rearfoot: 67.7 N·mm^− 1^) while providing the best energy absorption (forefoot: 14.0 J, rearfoot: 17.1 J) and return (forefoot: 11.5 J ≙ 82.3%, rearfoot: 14.4 J ≙ 84.4%). PN demonstrated the highest midsole compression stiffness (forefoot: 117.3 N·mm^− 1^, rearfoot: 94.4 N·mm^− 1^), with the lowest energy absorption (forefoot: 10.6 J, rearfoot: 13.1 J) and return (forefoot: 8.5 J ≙ 79.9%, rearfoot: 9.3 J ≙ 71.0%) among all standard models.

**Table 3:** Characteristics of the standard shoes

	AM		NA		PN	
	Forefoot	Rearfoot	Forefoot	Rearfoot	Forefoot	Rearfoot
Compression stiffness (N·mm^− 1^)	101.9	93.5	98.0	67.7	117.3	94.4
Maximum deformation (mm)	19.1	20.8	19.9	28.7	16.6	20.6
Energy absorption (J)	13.2	15.1	14.0	17.1	10.6	13.1
Energy return (j)	10.8	12.4	11.5	14.4	8.5	9.3
Energy return (%)	82.1	81.7	82.3	84.4	79.9	71.0
Mass (g)	200		224		225	

Shoes tested were in size US “men” 9 (NA, PN) and US “unisex” 9 (AM)

AM Asics Metaspeed Sky+, NA Nike Air Zoom Alphafly Next% 2, PN Puma Fast-R Nitro Elite v1

## Discussion

The aim of this study was to identify biomechanical factors associated with RE while wearing various types of AFT. In a cohort of 22 female and male trained long-distance runners, we found reduced GCT to be associated with lower eCoT. Simultaneously, we did not observe differences in RE between three standardized commercially available AFT models at the group level.

### Reduced GCT is Associated with Decreased eCoT

Our two-fold model selection strategy identified GCT as a robust predictor of eCoT (β = 0.025, 95% CI [0.010, 0.040]). Lower eCoT (i.e., improved RE) was associated with shorter GCT. Specifically, a 4 ms decrease in GCT corresponded to an approximate 1% reduction in eCoT (0.01 J⸱kg^− 1^⸱m^− 1^ decrease per 1 ms decrease in GCT). At first glance, these findings appear to contrast with those of van Hooren et al. [20], who reported a negative correlation between GCT and energetic cost when comparing an AFT with a traditional control shoe. However, such comparisons may be dominated by direct material effects of AFT, potentially obscuring biomechanical contributions. Notably, when comparing two AFT models with more similar material properties, their study showed that the shoe with lower GCT also exhibited lower energetic cost, which is consistent in direction with our within-athlete association and with the findings of Hunter et al. who showed a positive group-level effect of GCT for the AFT condition [7].

Moore et al. [39] reported a curvilinear (U-shaped) relationship between GCT and metabolic cost in a within-subject single-shoe design. They showed that trained runners self-select a GCT close to their mathematical optimum (within ~ 5%), such that deviations in either direction (with step frequency held constant) increased metabolic cost. The authors attributed this to a self-optimization that had been proposed previously [40]. This suggests that an individual optimum GCT may vary across runners and potentially across shoe conditions. In our data, AFT models differed significantly in GCT at the group level (p_ANOVA_ < 0.001), yet eCoT did not (p_ANOVA_ = 0.246), indicating that GCT is *one* of several interacting mediators (e.g., ankle and MTP kinematics, pelvis oscillation, step rate) and that these, together with direct material effects [18], may offset or obscure the effect of any single mediator at the group level.

To explore potential mechanisms, the results of our material tests (Table 3) provide additional context. Compared with PN, both AM and NA showed lower compression stiffness in the forefoot region alongside greater deformation and higher energy return. These more compliant and resilient midsole properties may lessen the mechanical demands of push-off and improve the efficiency of the calf, knee, and hip extensors [41]. Conceptually, elastic energy storage increases with force and decreases with stiffness (E ∝ F^2^/(2⸱k)); thus, a shorter GCT – often accompanied by higher peak forces – combined with lower forefoot stiffness could increase stored and returned energy in the leg-shoe system and reduce required active muscle work. However, these material differences did not translate into superior group-level RE, likely due to additional indirect and individual effects. Finally, footwear effectiveness also depends on the timing of energy return [42], which may be optimized in an athlete-specific manner through interactions between shoe properties and foot–ankle mechanics, potentially mediated by changes in GCT.

Since GCT and step rate are correlated, we tested whether the effect of GCT changed after adjusting for step rate in a model including both GCT and step rate. We found the GCT effect remained of similar magnitude. Together with the results of the sensitivity analysis, this supports GCT as a robust within-athlete predictor of eCoT differences among AFT models.

### Other Biomechanical Factors and Shoe Mass

Only step rate and MTP peak dorsiflexion angle showed some level of importance in our AICc-based model averaging approach, but were not present in the more conservative LASSO analysis. This could mean that discrete kinematic parameters do not suffice to reflect joint kinematic differences, resulting in changes in RE. Furthermore, previous research reported that shoe mass impacts RE, where for each 100 g of additional shoe mass per shoe, the metabolic rate was increased by ~ 1% [43]. In our study, we did not find shoe mass to be significantly associated with intra-individual differences in RE. The shoes tested in our study all had similar mass. Only AM was notably lighter (200 g vs. PN 225 g and NA 224 g). This alone results in approximately 0.25% increase in metabolic rate in NA and PN in the tested shoe size (US men’s 9). It is likely that other effects outweigh this apparent disadvantage or that it is lost within the measurement error of the metabolic cart.

### No Group-Level Differences in eCoT Between AFT Models

None of the AFT models tested here was superior in terms of RE at the group level. Rather than indicating a lack of effect, this finding highlights substantial inter-individual variability in response to different AFT models and suggests that no single shoe can be considered universally optimal. In line with this, Joubert and Jones [44] reported no statistically significant difference in oxygen uptake between AM and the first generation of the NA at 16.0 km⸱h^− 1^ (AM: 50.4 ± 1.7 ml⸱kg^− 1^⸱min^− 1^; first generation NA: 50.1 ± 1.9 ml⸱kg^− 1^⸱min^− 1^), which is fairly consistent with our findings (15.0 ± 2.3 km⸱h^− 1^; AM: 48.9 ± 6.3 ml·kg^− 1^·min^− 1^; NA: 48.9 ± 6.4 ml·kg^− 1^·min^− 1^). However, they also observed significantly worse economy for other AFT models. Within the Connick and Lichtwark framework [18], such disparities may reflect direct-pathway differences in material properties and/or indirect, runner–shoe interaction effects (biomechanical compatibility) in their cohort (*n* = 12).

### Future Perspective and Practical Implications

It has long been established that energy return from footwear needs to be effective at the right time, in the right direction, and in the right frequency [42, 45]. While shoe manufacturers have now substantially narrowed this gap, there will remain an individual component that cannot be optimized for every type of runner with a single AFT model. Additionally, some manufacturers might have superior materials compared with others. Nevertheless, it is likely that the gap in material technologies may be reduced in the future due to official regulations [46]. With respect to the Connick & Lichtwark framework [18], this would mean that the effects of the direct pathway (material property) will become less significant and that footwear individualization – in the sense of a runner-footwear matching – becomes more important. Depending on potential changes in the official regulations regarding individualized footwear, our findings can also guide developers of bespoke footwear using additive manufacturing technologies [47]. In the present study, we demonstrated that GCT is one biomechanical factor associated with intra-individual changes in eCoT when running with various AFT models. According to our results, a shoe leading to a 4 ms shorter GCT could decrease eCoT by 1%. Our shoe selection was based on the availability of three established running shoe manufacturers. Future research is warranted to investigate if the mediating effect of GCT on RE persists beyond these models.

### Limitations

There are certain limitations in the study design which must be addressed. Recent recommendations suggest using at least two trials per condition when investigating RE changes with AFT [48]. Measurement errors in metabolic analyzers can be significant, potentially leading to reversed interpretations of metabolic changes. Conducting two or more trials for all shoe conditions (up to six, including individual shoes), however, would have resulted in a running exposure of up to 60 min or more, potentially introducing fatigue effects. Additionally, the extended duration of the experiment could have substantially reduced participant responsiveness. In a subsequent analysis, we averaged the spiroergometric data over the last two instead of three minutes as a simple sensitivity analysis. Besides minor changes to model-averaged estimates, the p-values of the biomechanical predictor variables did not change, indicating robust results. Because the project was conducted in an applied context with fixed scheduling constraints, a second testing day was not feasible. Furthermore, the running bouts were conducted on a motorized treadmill, which can be considered more compliant than road running. It is often suggested to investigate RE when using AFT on rigid surfaces, i.e., overground or on a rigid treadmill [49, 50]. Since we were not able to quantify the surface stiffness of our treadmill, we cannot estimate the effect this had on our results. We used an individualized running speed based on the athletes’ season’s best times to simulate a marathon running pace. Other studies compared runners at standardized uniform running speeds. This limits the comparability of our findings to an extent; on the other hand, however, our approach offers better ecological validity. Lastly, our original a priori power analysis (RM-ANOVA, *n* = 32) does not directly apply to the revised mixed-effects, within-athlete design adopted after peer-review. We therefore emphasize estimates and CIs, model-comparison metrics, and sensitivity analysis rather than post-hoc power.

## Conclusion

The present investigation demonstrated that individual changes in GCT were associated with changes in RE when running with different types of AFT. Our within-subject analysis suggested that shoes that reduced runners’ GCT were associated with better RE. Our null result from the group level-analysis and the low variance explained by GCT in our within-subject model, on the other hand, showed that other mediating factors (in combination) influenced RE across various AFT models. Future studies should include material properties in combination with individual biomechanics and anthropometrics to investigate distinct interactions of athletes and footwear.

## Supplementary Information

Below is the link to the electronic supplementary material.

Fig. S1 Leave-one-participant-out sensitivity analysis of the coefficient estimates of ground contact time as the single fixed effect in the final linear mixed effects model. The black dashed vertical line and the shaded area are the result (coefficient estimate and 95% CIs) of the model containing all participants. The blue dots and horizontal bars are the coefficient estimates and 95% CIs of the model fit with the respective participant left out of the data set

## Data Availability

The datasets used and analysed during the current study are available from the corresponding author on reasonable request.

## Abbreviations

AFT: Advanced footwear technology
AM: Asics Metaspeed Sky+
BMI: Body mass index
CFP: Carbon fiber plate
CoM: Center of mass
eCoT: Energetic cost of transport
GCT: Ground contact time
HR: Heart rate
[La]: Blood Lactate concentration
LBS: Longitudinal bending stiffness
MTP: Metatarsophalangeal
NA: Nike air zoom alphafly next% 2
O_2_CoT: Calculated oxygen cost of transport
PN: Puma Fast-R Nitro Elite v1
RE: Running economy
RER: Respiratory exchange ratio
RF: Respiratory frequency
ROM: Range of motion
VIF: Variance inflation factor
VO_2_: Oxygen uptake
